# Activation Effects of Carnosine- and Histidine-Containing Dipeptides on Human Carbonic Anhydrases: A Comprehensive Study

**DOI:** 10.3390/ijms21051761

**Published:** 2020-03-04

**Authors:** Giulio Vistoli, Giancarlo Aldini, Laura Fumagalli, Clelia Dallanoce, Andrea Angeli, Claudiu T. Supuran

**Affiliations:** 1 Department of Pharmaceutical Sciences, University of Milan, Via Luigi Mangiagalli 25, 20133 Milan, Italy; Giancarlo.aldini@unimi.it (G.A.); laura.fumagalli@unimi.it (L.F.); Clelia.dallanoce@unimi.it (C.D.); 2 Department of Neurosciences, Psychology, Drug Research and Child Health, University of Florence Via U. Schiff 6, Sesto Fiorentino, 50019 Florence, Italy; andrea.angeli@unifi.it

**Keywords:** carbonic anhydrase, activator, proton shuttling, carnosine, histidine, dipeptide

## Abstract

l-Carnosine (β-Ala-l-His) and several other histidine-containing peptides, including two N-methylated forms on the imidazole ring (l-anserine and l-balenine), two derivatives modified on the carboxyl function (carcinine and l-carnosinamide), two analogues differing in the length of the N-terminal residue (l-homocarnosine and Gly-l-His) and the N-acetyl derivatives, were investigated as activators of four isoforms of the metalloenzyme carbonic anhydrase (CA, EC 4.2.1.1). The four human isoforms hCA I, II, VA and IX were activated in the low to high micromolar range, with a rather complex structure activity relationship. A performed computational study allowed us to rationalize these results and to propose a binding mode of these activators within the enzyme active site. Similarly to other CA activators, the here studied peptides could find relevant pharmacological applications such as in the management of CA deficiencies, for therapy memory and enhancing cognition or for artificial tissues engineering.

## 1. Introduction

The dipeptide l-carnosine (β-Ala-l-His) represents the prototype of a set of histidine-containing peptides which are found in the entire animal kingdom, where they are primarily expressed in the muscles and are significantly abundant also in other excitable tissues [1,2]. As shown in Table 1, they include two N-methylated forms on the imidazole ring (l-anserine and l-balenine), two derivatives modified on the carboxyl function (carcinine and l-carnosinamide), two analogues differing in the length of the N-terminal residue (l-homocarnosine and Gly-l-His) and the N-acetyl derivative [3,4]. The detailed physiological and biochemical roles of these derivatives are still to be completely clarified, even though some bioactivities can be argued from their physicochemical properties. Thus, the well-known properties of the imidazole ring render almost all these derivatives metal-chelators, antioxidants and endowed with a significant buffering capacity [5]. Apart from N-acetyl-l-carnosine, these dipeptides are also able to inhibit the protein carbonylation by quenching reactive carbonyl species (RCS) through a two-steps mechanism which involves both the primary amine and the imidazole ring and yields a final Michael adduct [6]. The buffering capacity is primarily ascribable to the ionization properties of the imidazole ring which shows a pK value (pK = 6.72) close to the physiological pH [7]. However and along with this clear and direct mechanism, also indirect mechanisms can contribute to the buffering capacity especially when considering that histidine analogues (such as histamine and histidine itself [8]) are reported to be potent activators of some isoforms of the human carbonic anhydrases (hCAs) which play a key role in modulating and maintaining the physiological pH [9]:

(1) $${\mathrm{EZn}}^{2+}—{\mathrm{OH}}^{-}+{\mathrm{CO}}_{2}⇌{\mathrm{EZn}}^{2+}—{{\mathrm{HCO}}_{3}}^{-}\overset{+H_{2}O}{⇄}{\mathrm{EZn}}^{2+}—{\mathrm{OH}}_{2}+{{\mathrm{HCO}}_{3}}^{-}$$

(2) $${\mathrm{EZn}}^{2+}—{\mathrm{OH}}_{2}⇌{\mathrm{EZn}}^{2+}—{\mathrm{OH}}^{-}+H^{+}\;(\mathrm{rate}\;\mathrm{determining}\;\mathrm{step})$$

In the presence of an activator molecule ‘A’, Equation (2) becomes (3), as in the enzyme-activator complex the proton transfer reaction is no longer intermolecular but intramolecular, and thus favored [3,10]:

(3) $${\mathrm{EZn}}^{2+}—{\mathrm{OH}}_{2}+A⇌[{\mathrm{EZn}}^{2+}—{{\mathrm{OH}}_{2}}^{+}—A]⇌\left[{\mathrm{EZn}}^{2+}—{\mathrm{HO}}^{-}—{\mathrm{AH}}^{+}\right]\;⇌{\mathrm{EZn}}^{2+}—{\mathrm{HO}}^{-}+{\mathrm{AH}}^{+}\;\text{enzyme-activator complexes}$$

The interest for CAs activators has remarkably increased [10,11,12,13] in the last few years since the CA activation mechanism has been elucidated by one of our groups [8]. Indeed, CA activators (CAAs) participate to the catalytic cycle (Equations (1) and (2) above), more exactly in the rate determining step of it, which consists in the formation of the zinc hydroxide species of the enzyme by the loss of a proton from an acidic enzyme species, which has the zinc ion coordinated by three His residues and a water molecule, as shown in Equation (2) [8,11]. The proton is transferred from the water molecule to the external medium, and in many CA isoforms this process is assisted by residue His64, the proton shuttle residue, located in the middle of the active site cavity [8,10,11,12,13]. In the presence of CAAs, the proton transfer is performed both through the canonical, His64 shuttling, and through and additional shuttling which involves the activator molecule, which generally possesses protonatable moieties with pKa values in the range of 6–8 [11]. This is achieved through the formation of enzyme-activator complexes, as shown above in Equation (3), in which the proton transfer is intramolecular (nor intermolecular as in Equation (2), and as thus, favored [8,10,11,12,13].

The first study reporting the l-carnosine effect on the hCA enzymatic activity dates back to 2002 when a set of derivatives incorporating arylsulfonylureido moieties were described as potent and selective hCA activators [14]. Since then, very few studies have investigated the role of the carnosine derivatives on hCA enzymes and all these studies were focused on synthetic compounds as exemplified by the halogenated analogues proposed by Saada et al. in 2014 [15], while the activity of the above described set of natural histidine containing dipeptides was never investigated in a comprehensive way.

**Figure 1:**
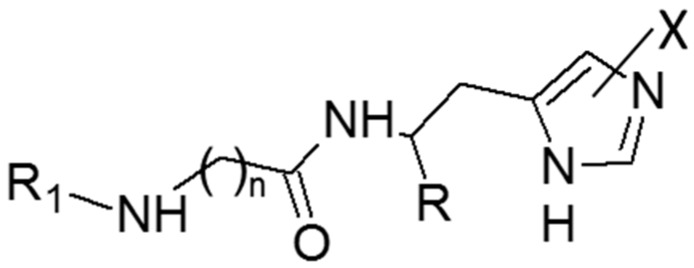
The general molecular formula of the tested compounds.

On these grounds, the present study was undertaken to systematically investigate the activating profiles of these histidine containing derivatives on a panel of representative human CA isozymes including hCA I, hCA II, hCA VA and hCA IX. As listed in Figure 1 and Table 1, the study comprised the above mentioned naturally occurring carnosine derivatives, two analogues including the non-natural D-His residue (i.e., d-carnosine [16] and d-carnosinamide), the tripeptide Gly-Gly-l-His, a well-known metal chelator [17], chosen here to investigate the capacity of enzymatic binding cavity to accommodate larger derivatives and finally a recently proposed synthetic analogue modified on the carboxyl group (i.e., l-carnosinol) [18]. The monitored bioactivities were finally rationalized by docking simulations involving the hCA II isozyme chosen due to the availability of resolved structures in complex with histidine [19].

## 2. Results

### 2.1. Biological Activity

Table 1 reports the activation constants (K_A_) for the here analyzed histidine containing derivatives as obtained by a stopped-flow CO_2_ hydrase assay on hCA I, hCA II, hCA VA and hCA IX isozymes. For a facile comparison, Table 1 includes also the activation constants for the reference compound histamine. A first general consideration involves the K_A_ averages for the four monitored CA isoforms (hCA I = 51.42 µM, hCA II = 93.85 µM, hCA VA = 25.41 µM and hCA IX = 23.94 µM) which reveal that the tested compounds showed an activation profile partly different compared to that of histamine. Indeed, histamine and carnosine analogues share a very modest activity on hCA II, while differing for the activity on the other three isozymes. In detail, histidine containing ligands show a good and similar activity on hCA VA and hCA IX and an overall poorer activity on hCA I, thus leading to a general activity trend, which can be schematized by: hCA IX ≅ hCA VA > hCA I >> hCA II.

A comparative analysis between the compiled K_A_ values reveals that there is no correlation between them (maximum r value equal to 0.48 between K_A_ values of hCA II and hCA VA) and this finding is clearly evident when considering that the most active compounds vary in the monitored isoforms and are: d-carnosinamide for hCA I, d-carnosine for hCA II, carcinine for hCA VA, and l-anserine for hCA IX which shows the best K_A_ value. The lack of correlations underlines that the activity of these derivatives, while sharing a common activating mechanism, is influenced by isozyme-specific factors, a finding which is in line with the above discussed activity trend.

A detailed analysis of the activity profiles for the tested compounds allows for some interesting considerations. First, d-carnosinamide, carnosinol and carcinine are more active on hCA VA compared to hCA IX, thus suggesting that modifications on the C-terminus, which neutralize the negative charge of the carboxyl group, can promote the hCA VA activity. Second, l-homocarnosine, Gly-Gly-l-His and N-acetyl-l-carnosine are more active on hCA IX compared to hCA VA, thus suggesting that increasing the steric hindrance of the N-terminus favors the interaction with hCA IX. Thirdly and apart from hCA II, for which all tested derivatives reveal very poor activities, the two methylated analogues (i.e., l-anserine and l-balenine) show very marked differences in all other isoforms although with different trends, since l-balenine is the more active regioisomer in hCA I, while l-anserine is more active on hCA VA and hCA IX. Such a remarkable regioselectivity emphasizes the pivotal role played by the imidazole ring in enhancing the catalytic activity as rationalized by the following docking simulations.

Finally and with regard to the studied enantiomeric pairs, Table 1 shows that d-carnosine is more active than l-carnosine in all tested isozymes with the hCA IX subtype being the most enantioselective one (ER = 4.6). As described by the following docking simulations, such an enantioselectivity can be explained by considering that the imidazole ring has to assume a well-defined arrangement to promote the enzymatic activity and this is clearly influenced by the histidine configuration. Based on these premises and considering the remarkable activity of l-carnosinamide on hCA IX, the synthesis of the d-carnosinamide was accomplished. Unfortunately, d-carnosinamide was found to be the eutomer on hCA I, roughly equipotent on hCA II and hCA VA, but markedly less active than l-carnosinamide on hCA IX (ER = 9.7). This unsatisfactory result can be rationalized by considering that the different steric hindrance of the residues contacting the C-terminus between the tested isozymes can alter the relation between activity and configuration as suggested by the following calculations.

### 2.2. Computational Study for the Binding of CAAs

**Figure 2:**
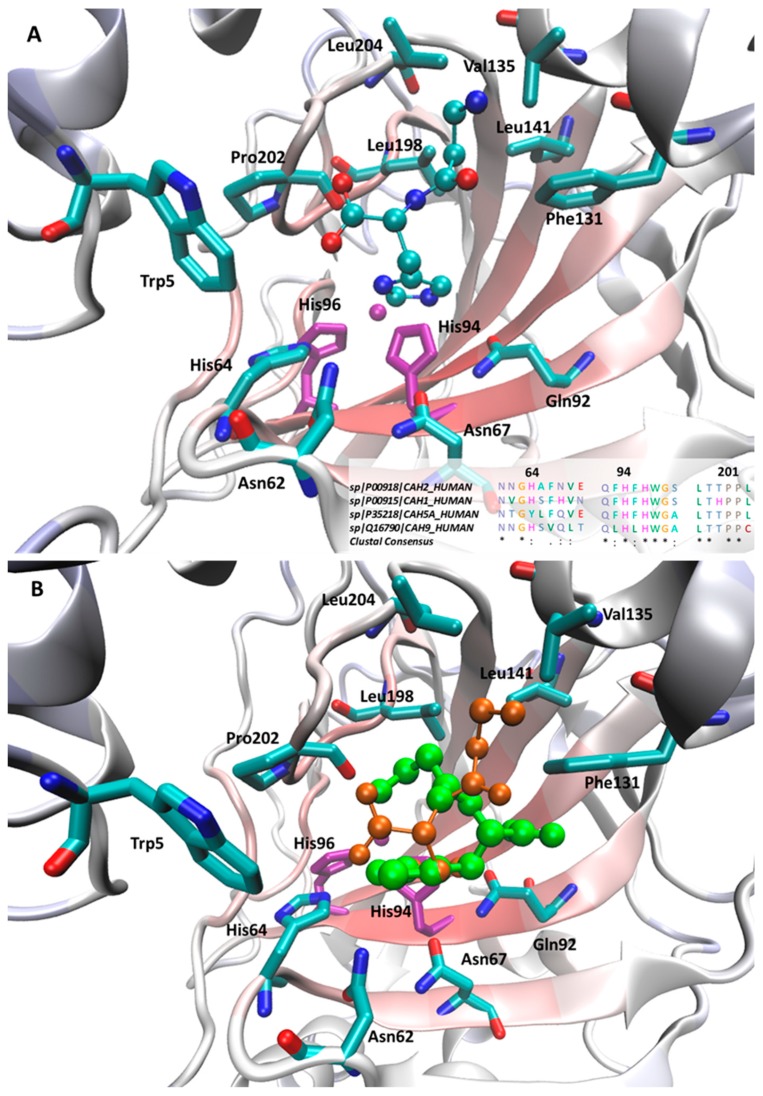
Main interactions stabilizing the putative complexes as computed for d-carnosine (**A**) and comparison between d-carnosine (in orange) and l-carnosine (in green) within the hCA II binding site (**B**). In both Figures, the zinc ion plus the coordinating histidine residues are depicted in purple. The inlet in Figure 2A shows the multiple sequence alignments (and the relative consensus) for the four tested CA isoforms as computed by ClustalX [21].

With a view to rationalizing the activity profiles described above, docking simulations were performed on the hCA II isoform. Even though the tested compounds show, on average, poor activities on this isozyme, the availability of resolved structures in complex with structurally related activators [19] allow the generation of reliable docking results, which can be utilized to extrapolate general hints in order to explain the observed differences between the tested ligands in the other isozymes. Figure 2A shows the putative complex for the most active d-carnosine within the CA II catalytic cavity and reveals that the ligand’s imidazole ring assumes a pose superimposable with that observed for the co-crystallized histidine and is surrounded by a set of interacting residues with which it can elicit π-π stacking plus H-bonds (such as Asn62, His64, Asn67, Gln92 and, to minor extent His94). The involvement of amide containing side chains seems to confirm the stabilizing role of stacking interactions between amide and heteroaryl moieties as recently investigated [20].

The key differences between d-carnosine and the resolved histidine concern the arrangement of the charged termini. Here, the carboxyl terminus is seen to contact Trp5 eliciting a weak H-bond, while the β-alanine portion assumes a folded geometry by which it contacts hydrophobic residues (Val135, Leu141, Pro202 and Leu204), with the ammonium head approaching Phe131 with which it can stabilizes charge transfer interactions. Since the interactions described above involve almost all d-carnosine moieties, one may explain the observed stereoselectivity between the two carnosine enantiomers, especially when considering the steric hindrance exerted by Trp5. Indeed, Figure 2B compares the best poses as computed for the two carnosine enantiomers and reveals that the charged termini of l-carnosine assume a swapped pose by which its imidazole ring is constrained to assume a more external arrangement compared to that seen for d-carnosine.

Overall, the described complexes are seen to tightly stabilize the arrangement of the imidazole ring, which is directly involved in the activating mechanism, while the charged termini seem to be engaged by weaker contacts and play a rather marginal role. This finding can explain why modifications on the charged moieties often exert beneficial effects. The positive effect can be also due to the breaking of the intramolecular ionic bridge, which negatively constrains the flexibility and the accommodation of the beta-alanine residue. Without this, the N-terminal tail increases its flexibility and facilitates the accommodation even of bulky derivatives as exemplified by N-acetyl-carnosine, which assumes more extended conformations by which its N-terminal portion approaches the residues lining the cavity rim, such as Trp5, Trp16, Phe20, Phe131, Val135, Pro202 and Leu204. Notably, Trp5 and more in general the above mentioned residues can explain the different sensitivity to the ligand size as observed for the considered isoforms. The greatest differences are observed in the first three residues since the Trp-Trp-Phe triad of hCA II is replaced by a similarly bulky Trp-Trp-Tyr triad in hCA I and hCA VA, while being clearly less bulky in hCA IX (Gly-Trp-Ser), thus justifying the good activity that bulky ligands, such as Gly-Gly-His and homocarnosine, show for hCA IX. By contrast, the hydrophobic region around Pro202 is highly conserved in all considered isozymes as depicted in the inlet of Figure 2A.

With a view to rationalizing the differences observed in the CA isozymes, the inlet in Figure 2A displays the sequence alignments between the four considered CA subtypes focusing attention on the residues primarily involved in the activator recognition. With regard to residues facing the imidazole ring, Figure 2A reveals that the residues Gln92, His94, His96, which are also involved in zinc chelation, are constantly conserved in all subtypes, while greater differences are seen in the residues around His64, which is conserved in all isoforms apart from CA VA where it is replaced by a tyrosine.

In detail, Asn62 and Asn67 show a larger variability, which can explain the different regioselectivity shown by the methylated analogues. Indeed, in all isozymes, hCA I excepted, anserine is more active than balenine and all isoforms show H-bonding residues in those positions apart from CA I which has an alkyl side chain (Val63), thus suggesting that the corresponding missed H-bond can induce the regioselectivity change observed in CA I. Ala 65 is replaced in hCA I and hCA IX by H-bonding residues and this can beneficially contribute to their interaction with the tested activators.

## 3. Discussion

To the best of our knowledge, this is the first study which investigates in a comprehensive way an extended set of naturally occurring histidine-containing peptides against a representative panel of therapeutically relevant hCA isoforms. The obtained results highlight that, apart from the CA II isoform, on which all imidazole derivatives show very poor activities, almost all tested molecules elicit significant activating effects which appear to be more marked on the hCA VA and hCA IX isozymes. As mentioned in the Introduction, various biological activities were reported for carnosine and its derivatives [22] and the still debated mechanisms were substantially related to their chemical properties and in particular to the specific characteristics of the imidazole ring which render such molecules antioxidants, buffers, metal-chelators and carbonyl quenchers [23,24]. All reported therapeutic effects were supposed to be an indirect consequence of the above cited chemical effects and until now very few direct mechanisms based on the carnosine interaction with specific targets were reported [25].

In contrast, this study underlines that the histidine containing peptides are able to significantly increase the activity of different carbonic anhydrases through a specific binding within their catalytic pockets. On these grounds, one may consider that the observed capacity of carnosine analogues to restore and maintain the physiological pH is not merely due to an intrinsic buffering capacity of the imidazole ring, but is also ascribable to the catalytic effect on CA isoforms. Clearly, the relation between buffering capacity and CA activation depends on the peptide concentration and the key relevance of such an enzymatic mechanism is that it may occur at concentrations far below than those required for a satisfactory buffering effect especially for the most active compounds. When considering the rapid hydrolysis that carnosine and some of its derivatives undergo in plasma by serum carnosinase, one may figure out that the buffering capacity has a predominant (but yet not exclusive) role in those tissues which synthesize carnosine at mM concentrations (such as skeletal muscles) [26] while the CA activation can have a relevant role in the other tissues which are reached by low (but non-negligible) amounts of histidine containing peptides [27]. In this context, the carnosine analogues which show a lower susceptibility to the carnosinase hydrolysis (such as homocarnosine and anserine) can have crucial effects [28].

Remarkably, such an activation effect on CAs allows for a better understanding of many reported carnosine biological activities. As an example, several studies reported significant beneficial effects of carnosine and its analogues on cognitive impairment [29,30] and the proposed mechanisms were substantially focused on antioxidant and metal-chelation properties of these peptides which elicit anti-inflammatory and anti-aggregant neuroprotective effects. [31] Since the CA activators are emerging for their capacity to enhance cognition and memory [10,11], the here reported results suggest that the beneficial effects of histidine containing dipeptides on brain activity can be also ascribed to their activating effects on CA isozymes.

## 4. Materials and Methods

### 4.1. Materials

l-Carnosine, d-carnosine and l-carnosinol were kindly provided by Flamma S.p.A. (Chignolo D’Isola, Bergamo, Italy). l-Carnosinamide, l-homocarnosine, l-anserine, carcinine, Gly-l-His, Gly-Gly-l-His and N-acetyl-l-carnosine were purchased from Merck KGaA (Darmstadt, Germany), while l-balenine was prepared following a previously described procedure [32].

### 4.2. Synthesis of d-Carnosinamide

**Scheme 1:**
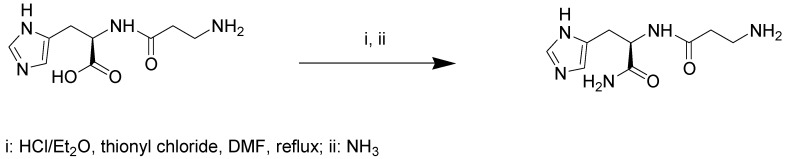
Synthesis of d-carnosinamide.

d-Carnosine (100.0 mg, 0.44 mmol) was dissolved in HCl/Et_2_O (1.3 N, 7.0 mL). The solution was cooled at 5 °C and then SOCl_2_ (0.1 mL, 1.32 mmol) was added dropwise. Subsequently the mixture was allowed to reach room temperature and DMF (2 mL) was added. Then the reaction mixture was heated at reflux overnight. Next, the mixture was cooled at 5 °C, saturated with NH_3_ and stirred overnight (Scheme 1). Afterwards the solid was removed by filtration, washed with cooled Et_2_O (3 × 2 mL) and dried under vacuum to obtain d-carnosinamide as a hygroscopic white solid (74.34 mg; 75% yield). H-NMR (300 MHz, D_2_O) δ (ppm): 7.48 (s; 1H); 6.68 (s; 1H); 4.38 (dd; 1H, X of His, *J*_XA_ = 8.3 Hz, J_XB_ = 6.0 Hz); 3.01 (t; 2H, Hz CH_2_ α to the NH_2_, *J* = 6.7 Hz); 2.90 (dd; 1H; B of His, *J*_AB_ = 15.0 Hz, *J*_BX_ = 6.0 Hz, 1H); 2.91 (dd; 1H; A of His, *J*_BA_ = 15.0, *J*_AX_ 8.3 Hz); 2.45 (m; 2H, CH_2_ in β to NH_2_).

### 4.3. Biological Assays

An Sx.18Mv-R Applied Photophysics (Oxford, UK) stopped-flow instrument has been used to assay the catalytic activity of various CA isozymes for CO_2_ hydration reaction [33]. Phenol red (at a concentration of 0.2 mM) was used as indicator, working at the absorbance maximum of 557 nm, with 10 mM Hepes (pH 7.5, for α-CAs) or TRIS (pH 8.3, for γ-CAs) as buffers, 0.1 M NaClO_4_ (for maintaining constant ionic strength), following the CA-catalyzed CO_2_ hydration reaction for a period of 10 s at 25 °C. The CO_2_ concentrations ranged from 1.7 to 17 mM for the determination of the kinetic parameters and inhibition constants. For each activator at least six traces of the initial 5–10% of the reaction have been used for determining the initial velocity. The uncatalyzed rates were determined in the same manner and subtracted from the total observed rates. Stock solutions of activators (at 0.1 mM) were prepared in distilled-deionized water and dilutions up to 1 nM were made thereafter with the assay buffer. Enzyme and activator solutions were pre-incubated together for 15 min prior to assay, in order to allow for the formation of the enzyme–activator complexes. The activation constant (K_A_), defined similarly with the inhibition constant K_I_, can be obtained by considering the classical Michaelis–Menten equation (Equation (4)), which has been fitted by non-linear least squares by using PRISM 3:

v = v_max_/{1 + (K_M_/[S])(1 + [A]_f_/K_A_)} (4)

where [A]_f_ is the free concentration of activator.

Working at substrate concentrations considerably lower than K_M_ ([S] << K_M_), and considering that [A]_f_ can be represented in the form of the total concentration of the enzyme ([E]_t_) and activator ([A]_t_), the obtained competitive steady-state equation for determining the activation constant is given by Equation (5):

v = v_0_.K_A_/{K_A_ + ([A]_t_ − 0.5{([A]_t_ + [E]_t_ + K_A_) − ([A]_t_ + [E]_t_ + K_A_)^2^ − 4[A]_t_.[E]_t_)^1/2^}} (5)

where v_0_ represents the initial velocity of the enzyme-catalyzed reaction in the absence of activator [34,35,36,37].

### 4.4. Molecular Modelling

The conformational profile of the tested activators was explored as explained in previous studies [38]. Even though the induced CA-II activation is very weak, docking simulations involved this isoform due to the availability of resolved structures in complex with activators. Indeed and due to the satisfactory degree of conservation of the residues involved in the activator’s binding site (see Figure GV1C), these resolved CA-II complexes can be used as a general platform to rationalize the obtained results for all the evaluated isoforms. In detail, docking simulations involved the resolved CA-II structure in complex with L-histidine (PDB Id: 2ABE), a known activator similar to those here considered [17]. The CA structure was prepared by removing water molecules (apart from that bound to zinc ion) and crystallization additives. After preliminary checks to avoid gaps or unphysical occurrences, the enzyme structure was then minimized by keeping fixed backbone atoms plus zinc ion and chelating residues to preserve the resolved folding. The so optimized CA-II structure was finally used in docking simulations after removing the bound activator. In detail, docking calculations were based on the PLANTS program and focused onto a 10 Å around the bound (and deleted) activator [39]. For each tested ligand, 10 poses were generated and scored by the ChemPLP function with a speed equal to 1. Even without simulating explicitly the network of water molecules which connects the activator to zinc ion and which is required for the activation mechanism, the generated poses were also selected by considering the distance between the ligand’s imidazole ring and the zinc ion which should fall around 7.5 Å as seen in the resolved structure.

## 5. Conclusions

In summary, the present study investigated the activating effects of an extended set of histidine containing peptides on therapeutically relevant hCA isozymes revealing their significant activities on all tested hCA subtypes apart from hCA II on which all histidine analogues are poorly active. SAR analysis and docking simulations emphasize the key role of the imidazole ring as well as, to minor extent, of the C-terminus, while the N-terminal residues appears to play a more marginal role affecting at most the subtype-selectivity. The here reported activities allow for an in depth re-evaluation of many biological activities ascribed to carnosine and its derivatives and, more generally, these compounds appear to be valuable candidates for the various applications recently emerged for the CA activators, such as in the management of CA deficiencies, for therapy memory and enhancing cognition and for artificial tissues engineering based on bioinorganic scaffolds.

## Figures and Tables

**Table 1:** Considered carnosine derivatives **1**–**10** and their CA activating properties against human CA isoforms hCA I, II, VA and IX, by a stopped-flow CO_2_ hydrase assay. Histamine **11** was used as reference standard activator.

No.	Compound	R	R_1_	X	n	K_A_ (µM) *			
						hCA I	hCA II	hCA VA	hCA IX
**1**	l-carnosine	COOH	H	H	2	92.1	83.4	17.2	46.3
**2**	d-carnosine	COOH	H	H	2	54.3	76.6	8.93	10.1
**3**	l-carnosinamide	CONH_2_	H	H	2	45.2	91.5	18.5	4.71
**4**	d-carnosinamide	CONH_2_	H	H	2	16.6	>100	16.1	45.5
**5**	l-homocarnosine	COOH	H	H	3	77.0	88.9	51.3	7.35
**6**	l-anserine	COOH	H	1-Me	2	80.4	93.7	17.4	1.14
**7**	l-balenine	COOH	H	3-Me	2	20.7	>100	47.2	50.2
**8**	carcinine	H	H	H	2	16.6	>100	6.4	38.0
**7**	Gly-l-His	COOH	H	H	1	31.9	90.1	9.83	11.5
**8**	Gly-Gly-l-His	COOH	Gly	H	1	74.3	>100	52.8	19.4
**9**	N-acetyl-X-carnosine	COOH	Acetyl	H	2	79.6	102	47.1	32.6
**10**	l-carnosinol	CH_2_OH	H	H	2	28.3	>100	12.2	20.5
**11**	histamine	-	-	-	-	2.1	125	0.010	35.1

* Mean from three different assays (errors in the range of ± 10% of the reported values, data not shown).

## Abbreviations

CA	Carbonic Anhydrase
ER	Eudismic Ratio
RCS	Reactive Carbonyl Species
SAR	Structure-Activity Relationships
